# Analysis of Different Ploidy and Parent–Offspring Genomic DNA Methylation in the Loach *Misgurnus* *anguillicaudatus*

**DOI:** 10.3390/ijms17081299

**Published:** 2016-08-22

**Authors:** He Zhou, Tian-Yu Ma, Rui Zhang, Qi-Zheng Xu, Fu Shen, Yan-Jie Qin, Wen Xu, Yuan Wang, Ya-Juan Li

**Affiliations:** 1 Key Laboratory of Mariculture and Stock Enhancement in North China’s Sea, Ministry of Agriculture, Dalian Ocean University, Dalian 116023, China; zhouhe@dlou.edu.cn (H.Z.); 15712359285@163.com (T.-Y.M.); 15804763943@163.com (R.Z.); 18255258497@163.com (Q.-Z.X.); qin_tina@163.com (Y.-J.Q.); xuwen19891022@163.com (W.X.); 13591775473@163.com (Y.W.); 2 Fisheries Technology Extension Station of Beijing, Beijing 101105, China; dearhoodoo@163.com

**Keywords:** *Misgurnus anguillicaudatus*, diploid, triploid, tetraploid, parent–offspring generation, DNA methylation

## Abstract

In this study, we selected natural polyploidy loach (diploid, triploid and tetraploid) and hybrid F_1_ generation obverse cross (4 × 2) and inverse cross (2 × 4) by diploids and tetraploids as the research model. The MSAP (methylation-sensitive amplified polymorphism) reaction system was established by our laboratory to explore methylation levels and pattern diversification features at the whole genome level of the polyploidy loach. The results showed that the total methylation and full methylation rates decreased on increased ploidy individuals; moreover, the hemimethylation rate showed no consistent pattern. Compared with diploid loach, the methylation patterns of tetraploid sites changed 68.17%, and the methylation patterns of triploid sites changed 73.05%. The proportion of hypermethylation genes is significantly higher than the proportion of demethylation genes. The methylation level of reciprocal cross F_1_ generation is lower than the male diploid and higher than the female tetraploid. The hemimethylation and total methylation rate of the cross hybrid F_1_ generation is significantly higher than the orthogonal F_1_ generation (*p* < 0.01). After readjusting, the methylation pattern of genome DNA of reciprocal hybrids changed 69.59% and 72.83%, respectively.

## 1. Introduction

In the process of biological evolution, polyploidy (the duplication of the whole genome) is a universal and natural phenomenon. It leads to an increase of gene dosage and genome, which provides the space and potential for biological evolution. Polyploids generally exist among many plants and animals. Especially in freshwater fish, more than 30 kinds of polyploidy types have been found in China [1,2]. Researches in recent years show that the process of polyploidy and stabilization can lead to comprehensive changes in the structure, expression and function of genes. It affects the processes and the mechanisms of all genetic and epigenetic products [3]. Epigenetics is the genetic change in gene expression that does not involve the occurrence of DNA sequence change, which has important effects on the formation and evolution of multiples. The phenomenon and mechanism of epigenetics is researched widely and related to the polyploidy incorporation of DNA methylation, gene state, nucleolar dominance, etc. [4]. DNA methylation as a sort of modification process and reaction that commonly occurs in cells is a major epigenetic modification of genome DNA. It is also an important means of regulating gene function [5]. In recent years, methylation-sensitive amplified polymorphism (MSAP) technology has widely been used in animal and plant genome DNA methylation level and pattern analysis. However, there are few reports on the application in fish [6]. Loach (*Misgurnus* *anguillicaudatus*), besides being unique fish with high economic value, has multiple reproduction patterns and a phenomenon of ploidy variation [7]. Research shows that natural diploid, triploid, and tetraploid loaches exist in the same area in China [8,9,10]. Therefore, loach is an ideal material to explore the mechanism of homologous polyploids and allopolyploids. In the last 10 years, our laboratory has devised systematic research into the distribution, origin and formation mechanism of China’s natural polyploidy loach and has investigated the cellular and molecular patterns. China’s unique natural tetraploid loach is a homologous polyploidy in which both male and female are fertile [11,12,13]. At the same time, we also used natural tetraploid and diploid loach to hybridize a new triploid to research the composition of the gamete chromosomes [14]. To the best of our knowledge, there is no report into the epigenetic mechanism among different polyploid loach, hybrid triploid loach and parents. Therefore, in this research, we selected different polyploid loach (diploid, triploid and tetraploid) and a reciprocal cross generation of diploid and tetraploid parents as subjects. Our laboratory has established an MSAP (methylation-sensitive amplified polymorphism) reaction system to explore the changes on DNA methylation levels and patterns of different polyploidy and parent–offspring loaches, and to evaluate the regulation mechanism at gene expression changes of polyploidy in loach. This study aims to explore homologous and heterologous polyploidy mechanisms in fish.

## 2. Results

### 2.1. Ploidy Identification of Loach

**Figure 1:**
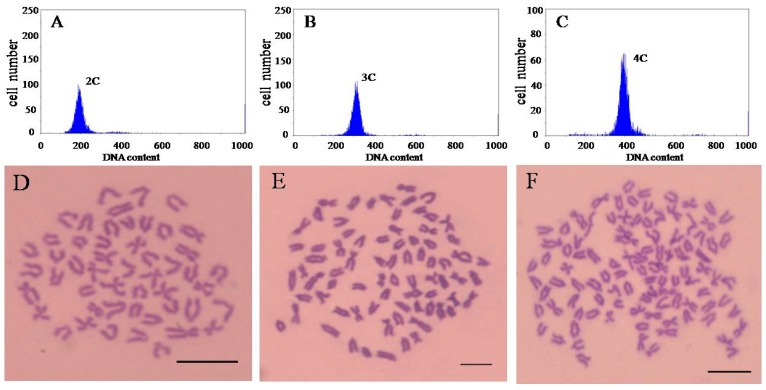
DNA-content flow-cytometrical histograms (**A**–**C**) and chromosomes (**D**–**F**) of diploid (**A**,**D**), triploid (**B**,**E**), and tetraploid (**C**,**F**) loach *Misgurnus* *anguillicaudatus*. Flow-cytometrical histograms showing: a diploid cell population with 2C DNA content (**A**); a triploid cell population with 3C DNA content (**B**); and a tetraploid cell population with 4C DNA content (**C**); The *Y*-axis denotes cell numbers and *X*-axis denotes channel numbers in each graph. Metaphase spreads of: a diploid cell with 50 chromosomes (**D**); a triploid cell with 75 chromosomes (**E**); and a tetraploid cell with 100 chromosomes (**F**). Scale indicates 10 μm.

Flow cytometry analysis indicates that the diploid, triploid and tetraploid cell population DNA content is 2C DNA (Figure 1A), 3C DNA (Figure 1B) and 4C DNA (Figure 1C), respectively. The ratio of DNA content in individual blood cells of diploid, triploid and tetraploid is 1:1.5:2. We used the chromosome counting method for further determination of ploidy in loaches because it is currently the most direct and accurate assay method. Chromosome observation showed that the chromosomes number is 2*n* = 50 in diploid (Figure 1D), 3*n* = 75 in triploid (Figure 1E) and 4*n* = 100 in tetraploid (Figure 1F).

### 2.2. The Levels of Genomic DNA Methylation in Different Ploidy of Loaches

The total bands and DNA methylation level, obtained by MSAP of diploid, triploid and tetraploid loaches, are shown in Table 1. The results show 5892 total bands from the selective amplification of eight primer pairs. Each pair of primers could amplify an average of 737 bands. There was a difference in methylation level between different ploidy loaches. Total methylation rate from high to low was diploid, 41.21% (full methylation 23.50% and hemimethylation 17.71%); triploid, 38.78% (full methylation 21.02% and hemimethylation 17.76%); and tetraploid, 37.03% (full methylation 22.22% and hemimethylation 14.81%). The results indicate a ploidy effect occurs with an increase of ploidy, the total methylation and full methylation ratio gradually reduced, while there is no specific rule in hemimethylation ratio. Statistical analysis showed that there was a significant difference in methylated level between triploid, tetraploid and diploid (*p* < 0.05), while there was no significant difference between triploid and tetraploid loaches (*p* > 0.05).

### 2.3. The Pattern of Genomic DNA Methylation on Different Ploidy for Loaches

We divided the pattern of DNA methylation of different ploidy loaches using the method of Bian [15], which brings amplification bands between different ploidy in loquat into four types as follows: A-type is a monomorphic site with the same methylation status between two ploidies, that is, both ploidies are hemimethylated or fully methylated; B-type is a demethylation type in which methylation exists in a control sample while the ploidy of loquat has a variation of demethylation in this site; C-type is an over- or hypermethylation type, the methylation level of some ploidy loquats were higher than the control; and D-type is the methine type, where the methylation level of some ploidy loquats are lower than the control. The four types from Bian [15] in our experiment are summarized as follows (Table 2): A-type is a monomorphic site. The DNA methylation level is the same among the different ploidy loaches. B-type is a demethylation type. Methylation occurred in the diploid, but in the triploid and tetraploid demethylation variation occurred at this site. C-type is the over- or hypermethylation type. Triploid and tetraploid methylation is higher than diploid. D-type is the methine type. Triploid and tetraploid methylation is lower than diploid, but there still exists a methylation status. The results of this study showed that compared with the diploid, the triploid’s DNA had 73.05% patterns of methylation variation; and the tetraploid’s DNA had 68.17% patterns of methylation variation (Table 2). Polymorphic sites in loaches with different ploidy show that the over- or hypermethylation type (C-type) was the highest, followed by the demethylation type (B-type), and sub-methylated type (D-type) is characterized as the lowest. This shows that many adjustments occur in loach methylation patterns, mainly based on over- or hypermethylation.

### 2.4. Level of Genomic DNA Methylation in Parents–Offspring of Loaches

The results of the amplification with the eight primer pairs show that the DNA methylation level of F_1_ was between their parents. It was lower than the male (female) diploid, but higher than the female (male) tetraploid. Using statistical analysis, the result of positive hybridization (4 × 2) showed that the female tetraploid, the male diploid and their offspring have significant differences in full methylation level (*p* < 0.01) and the male diploid and their offspring have no difference (*p* > 0.05). Comparing the female tetraploid and their offspring with the male diploid, there was a significant difference in hemimethylation level (*p* < 0.01). There is no difference between the female tetraploid and their offspring (*p* > 0.05). There was a significant difference in total methylation level (*p* < 0.01). The result in the hybrid (2 × 4) showed a significant difference between the female diploid and the male tetraploid and their offspring (*p* < 0.01) (Table 3). There is no significant difference in full methylation level among the F_1_, but there is significant difference in total methylation level and hemimethylation among the F_1_ (Table 4).

### 2.5. The Pattern of Genomic DNA Methylation in Parents-Offspring of Loaches

The diploid, tetraploid and their F_1_ offspring have been adjusted again for methylation pattern. They were divided into four types named A, B, C, and D (Table 5). A-type accounted for 30.41% and 27.17% of total methylation sites of orthogonal and anti-cross F_1_ generation. B-type accounted for 17.04% and 17.36% of total methylation sites of orthogonal and anti-cross F_1_ generation. C-type accounted for 39.33% and 39.95% of total methylation sites of orthogonal and anti-cross F_1_ generation. D-type accounted for 13.22% and 15.52% of total methylation sites of obverse and inverse F_1_ generation. Thus, the orthogonal F_1_ generation genomic DNA hypomethylate pattern had 69.59% mutations (demethylation into 17.04%, over- or hypermethylation into 39.33%, hypomethylation into 13.22%); anti-cross F_1_ hybrids genomic DNA also has 72.83% methylation pattern mutations (demethylation into 17.36%, over- or hypermethylation into 39.95%, methylene into 15.52%). Orthogonal or anti-cross hybrids are the main hypermethylation (Table 5).

## 3. Discussion

After species polyploidy, homologous polyploids and allopolyploids overcome the effects of genome doubling, some genetic and epigenetic changes will soon be produced, making it faster and better adapted to the new environment. The variation of epigenetics plays an important role in improving the diversity of polyploid gene expression, inducing the diploidization of genetics and cytology and promoting mutual coordination between the genome, and so on [16]. As an important form of epigenetic modifications, DNA methylation plays an important role in controlling gene expression, maintaining the stability of the genome, etc. Much research shows that after genome polyploidy the changes of levels and the adjustment of DNA methylation patterns is closely related to maintaining the stability of the genome, and balancing the reconstruction of nucleoplasm in polyploidy [17,18,19]. Generally speaking, DNA methylation and gene expression show a negative correlation, no matter whether in diploid or polyploid, DNA demethylation can lead to gene activation or translocation activation. High levels of methylation always lead to certain gene silencing or inhibition, expression and the expression level of related gene are likely to change [20]. In studies of *Arabidopsis thaliana* [21,22], when the genome DNA is at high level of methylation, it has been discovered that the expression of some genes are silenced. When methylation of DNA is processed, the level of methylation is reduced, and the related gene silencing phenomena is then lifted [19]. As a result, the relation of ploidy and DNA methylation levels in the previous are not the same. There are three main trends: first, along with the increase of ploidy, DNA methylation levels gradually increase, which shows a positive correlation [16,23]; second, along with the increase of ploidy, DNA methylation levels gradually reduce, which show a negative correlation [24,25]; and, third, no specific rule is shown [26,27,28].

In this research, DNA methylation levels of different ploidy also change; the total methylation rates of diploid, triploid and tetraploid were 42.21%, 38.78% and 37.03%, respectively. Methylation levels show a significant difference between triploid, tetraploid and diploid loach (*p* < 0.01). However, there was no significant difference between the triploid and tetraploid loach. This indicates that in the process of loach doubling its genome DNA methylation modification changes have taken place. The study found that the polyploid methylation levels did not increase with increasing polyploid levels, but gradually decreased with the increase of polyploid levels. This belongs to the second type of characteristic. After loach polyploidy, because of the increased genome, the methylation level is relatively lower, which means that some gene silencing is lifted, then related transcription can be reactivated. This means that the polyploid loach can regulate the redundant genes by DNA methylation. It also indicates that auto tetraploid loach has greater improvement potential and wider adaptability, shows advantageous characteristics of quick growth, low oxygen consumption rate, high nutritional value, etc. [29]. Many adjustments of polyploid methylation patterns can induce the activation and silencing of certain genes, leading to changes of epigenetics, making the polyploid better adapted to the needs of the environment. In this experiment, based on analyzing changes of different ploidy on loach genomic DNA methylation patterns, it was found that, compared with the diploid loach, methylation patterns of tetraploid sites have changed 68.17% (hypermethylation 36.84%, demethylation 26.80%, and hypomethylation 4.53%), and the triploid sites have changed 73.05% (hypermethylation 45.34%, demethylation 23.80%, and hypomethylation 3.91%). It can be seen that the proportion of hypermethylation gene is significantly higher than the proportion of demethylation. Polyploid loach, possibly through methylation of some functional genes, does not express to mitigate the effects of genome double dose effect. In research with wheat [30], rice [26], and *Stevia rebaudiana* [25], conclusions are basically identical.

Triploid fish have characteristics of infertility, fast growth, good meat quality, disease resistance etc.; they also have great significance in breeding [31]. An increasing number of studies show that the regulation of genetic level is conducive to the stability of hybridization and the genome doubling polyploid. Furthermore, the epigenetic modification, in which DNA methylation is the main mechanism, is very important for species formation and the successful evolution of multiple hybrid organisms. With the deepening research of DNA methylation, there are some reports about the relationship between heterosis and DNA methylation in plants. Romagnoli et al. [32] first put forward the relationship between heterosis and gene expression in crops, and found that 33% of specific expression products re-express. The study of triploid loquat and its parent by Wang [33] shows that the change of DNA methylation can promote the formation of triploid loquat heterosis. The study of hybrid larch species by Li et al. [34] shows that the formation of heterosis is related to the significant increase of DNA methylation in the offspring. The study of dandelion by Verhoeven et al. [35] compared MSAP and amplified fragment length polymorphism (AFLP) fragment inheritance in a diploid and triploid cross, and revealed de novo methylation variation between triploid F_1_ individuals. However, research in aquatic animals is relatively deficient. The research of DNA methylation patterns in red crucian carp and allotetraploid crucian carp through the MSAP method used by Song et al. [36] showed that allotetraploid crucian carp inherit 61.69% of their methylation pattern from both parents or one of them, indicating that the methylation level mainly follows the laws of Mendelian inheritance and maintains stable methylation patterns in tetraploid generations. The hybrid progeny of Zhi Kong scallops and Japanese scallops have 19.98% of sites in the methylation state, as reported by Yu et al. [37]. Cao et al. [6], through MSAP analysis of 20 full-sib in grass carp, found that methylation sites accounted for 75.9% of total sites. For triploid loach, whether it is a naturally occurring or synthetic, in the process of triploid formation from different individual genome reorganization and double phenomenon, to regain the balance of the gene of unpaired chromosomes, it cannot through large-scale chromosomal evolution, restore the chromosome diploidy in a short period of time. Therefore, it may regulate the extra genes and turning on and off gene expression through the changes of DNA methylation level and pattern. This study of methylation level and pattern analysis of diploid and tetraploid loach and reciprocal cross progeny shows that DNA methylation level and pattern of reciprocal cross F1 generation have obvious changes to parents. The DNA methylation level of hybrid F1 generation is between its parents, less than male parent diploid, and higher than female parent tetraploid. The hemimethylation and total methylation of reciprocal cross F1 generation is significantly higher than the orthogonal generation (*p* < 0.01). In individual detection, we found that all methylation rates are greater than the hemimethylation rates in most genomes of loach. Again, the loach methylation mode is given priority to full methylation. The result that all methylation rate is higher than hemimethylation rate in mammalian genomes are the same findings of Tang et al. [38]. The methylation pattern of reciprocal cross F1 generation have four types, namely monomorphism, demethylation, hypermethylation and hypomethylation, but they are mainly composed of type hypermethylation. This conclusion is basically identical to the research conclusion of Wang [33], but is not the same finding in corn, *Arabidopsis* *thaliana* [12,39], larch [34] and rice [40]. Related studies [34] have shown that methylation means that genes translate from activation to suppression; however, demethylation means that genes translate from suppression to activation. Whether methylation or demethylation is good for heterosis, different sites of enhanced or reduced methylation have a different effect on heterosis.

## 4. Materials and Methods

### 4.1. Ethics Statement

This study was performed according to the Guide for the Care and Use of Laboratory Animals in Dalian Ocean University, Dalian, China. All animal experiments comply with Chinese laws, regulations and ethics.

### 4.2. Materials

Thirty diploid loaches from farmers market were used as samples in Dalian, Liaoning Province, China. All natural triploid and tetraploid loaches were from Honghu, Hubei Province, China. Sixty individuals include 30 triploid loaches and 30 tetraploid loaches. All of the loaches were fed in aquaria (22 ± 1 °C) in the laboratory of Dalian Ocean University.

### 4.3. Ploidy Identification

In order to ensure the ploidy of loaches used in this experiment. The flow cytometry (Partec PAS-III, PARTEC, Münster, Germany) and chromosome number counts are used to observe ploidy detection. The specific method is as followed.

#### 4.3.1. Flow Cytometer

Blood was collected from the caudal peduncle of loaches, and stained by DAPI (4′,6-diamidino-2-phenylindole). Using the blood DNA content of diploid loaches as a normal diploid standard, each blood sample was measured separately.

#### 4.3.2. Preparation of Chromosome Samples

Loach was intraperitoneally injected with phytohemagglutinin (PHA; 6 μg·g^−1^ body weight), followed by a second PHA treatment 18–20 h later. At 4–6 h after the second PHA treatment, 0.1% colchicine (6 μg·g^−1^ body weight) was administered intraperitoneally. At 2–3 h after colchicines treatment, the animals were euthanized, and branchia tissue samples were collected. The branchia samples were treated under hypotonic stress in 0.8% sodium citrate for 40–45 min. The samples were fixed for 45 min in Carnoy’s solution (methanol: acetic acid = 3:1). The chromosome samples were prepared by the cold drop method and dyed by Giemsa. Statistical chromosome numbers were observed using optical microscopy.

### 4.4. Artificial Induce Spawning and Insemination

The parents were chosen from good development of gonad diploid and natural tetraploid loaches, injected with human chorionic gonadotropin (HCG) (injection dose: female, 20 to 25 IU·g^−1^; male, 10 to 12.5 IU·g^−1^). After 12 h, gently press female tetraploid loach abdomen, the eggs were discharged and collected in a 9 cm culture dish. Extrusion to genital pore on both sides along the male body made semen discharge. Semen was collected in centrifuge tubes by capillary (diluted 100 times with fresh water physiological saline). Using dry fertilization, hybridized combinations were obverse cross (4 × 2) and inverse cross (2 × 4). During the incubation and breeding period, the temperature was maintained at 25 ± 1 °C. Water was aerated, and dead fry were removed timely from the nursery pond. Fresh air and circulation was maintained in the breeding room.

### 4.5. DNA Extraction

Genomic DNA from loach fin tissue (there were 20 diploid, triploid and tetraploid loaches; 20 female and male loach of hybrid F1 generation; and four parents from obverse and inverse cross generations) was extracted by the SS-Phenol extraction method. The method was as follows. Around 0.2 g tail fin tissue was put it into 400 μL urea buffer (0.1 mol·L^−1^ Tris-HCl, pH: 7.5) with 10 μL proteinase K in buffer. Fin was digested for 12 h at 37 °C in a thermostat water bath. Digested fin was extracted once by saturated phenol, twice by phenol, chloroform, isoamyl alcohol mixture (volume ratio: 25:24:1), and once by chloroform. DNA was precipitated 30 min by 100% ethanol and centrifuged 10 min at speed of 12,000 rpm. The precipitation was dissolved in TE buffer (pH: 8.0) of 100 μL. The quality and concentration of DNA was tested by 1% agarose gel electrophoresis and nucleic acid concentration meter (Eppendorf Bio-Photometer D30, Eppendorf AG, Hamburg, Germany). The concentration of the various samples was adjusted for consistency, and then samples were stored at −20 °C.

### 4.6. Methylation-Sensitive Amplified Polymorphism (MSAP) Analysis

MSAP analysis of loach was by the MSAP technique reaction system [41] set up by this laboratory.

### 4.7. Enzyme Cleavage and Adaptor Ligation

Genomic DNA was enzyme cleaved by the endonuclease *Eco*R I/*Hpa* II and *Eco*R I/*Msp* I. A total of 20 μL enzyme cleavage reaction volume included: 800 ng of genomic DNA, 4 μL of 10× buffer Tango, 10 μL of *Eco*R I, and 10 μL *Hpa* II or *Msp* I. The reagents were thoroughly mixed and incubated 8 h at 37 °C. The adaptor ligation reaction comprised: 17 μL of enzyme cleavage product, both 1.5 μL of *Eco*R I and H-M adaptor (Table 6), 2 μL T4 DNA ligase (Transgen Biotech, Beijing China), 6 μL of 5× T4 buffer, and double-distilled water to 30 μL. Thoroughly mix reagent and incubate ligation for 2 h at 22 °C.

### 4.8. Preselective Amplification

The pre-amplification reaction consisted of: 4 μL adaptor ligation product, both 0.8 μL of primers E-A (10 mmol·L^−1^) and H-M (10 mmol·L^−1^) (Table 6), and 1.6 μL of each dNTP (2.5 mmol·L^−1^), 2 μL 10× PCR buffer (Mg^2+^ free), 1.2 μL MgCl_2_ (2.5 mmol·L^−1^), 0.2 μL *Taq* DNA polymerase (5 U·μL^−1^), and double-distilled water to 20 μL. PCR conditions were as follows: initial denaturation for 2 min at 94 °C, followed by 30 cycles of denaturation for 30 s at 94 °C, annealing for 40 s at 56 °C and extension for 60 s at 72 °C, and final extension for 60 s at 72 °C. Product was temporary stored at 4 °C. The quality and concentration of pre-amplification product was tested by 1% agarose gel electrophoresis. Pre-amplification product was diluted 20 times and used as selective amplification template. Residual product was stored at −20 °C. Adaptor and primer combinations are shown in Table 6.

### 4.9. Selective Amplification

Pre-amplification product was diluted 20 times in double-distilled water and was used as the template for selective amplification. The selective amplification reaction consisted of: 2 μL diluted pre-amplification product, both 1.5 μL of E (10 mmol·L^−1^) and H (10 mmol·L^−1^) (Table 6), 1.5 μL of each dNTP (2.5 mmol·L^−1^), 3 μL of 10× PCR buffer (Mg^2+^ free), 1.2 μL of MgCl2 (2.5 mmol·L^−1^), 0.2 μL *Taq* DNA polymerase (5 U·μL^−1^), and double-distilled water to a final volume of 20 μL. PCR conditions were as follows: initial denaturation for 2 min at 94 °C, followed by 12 cycles of denaturation for 30 s at 94 °C, annealing for 40 s at temperature from 65 to 56 °C (each cycle reduces in 0.7 °C increments) and extension for 60 s at 72 °C, followed by 30 cycles of denaturation for 40 s at 94 °C, annealing for 40 s at 56 °C and extension for 60 s at 72 °C, and final extension for 60 s at 72 °C. Product was temporary stored at 4 °C. Formamide loading buffer (10 μL) was added to the selective amplification product and the mixture was denatured for 5 min at 94 °C followed by incubation in on ice to denature the product immediately. The denatured product was tested by polyacrylamide gel electrophoresis (PAGE) and silver nitrate dying gel.

### 4.10. MSAP Bands Statistics, Analysis Method

**Figure 2:**
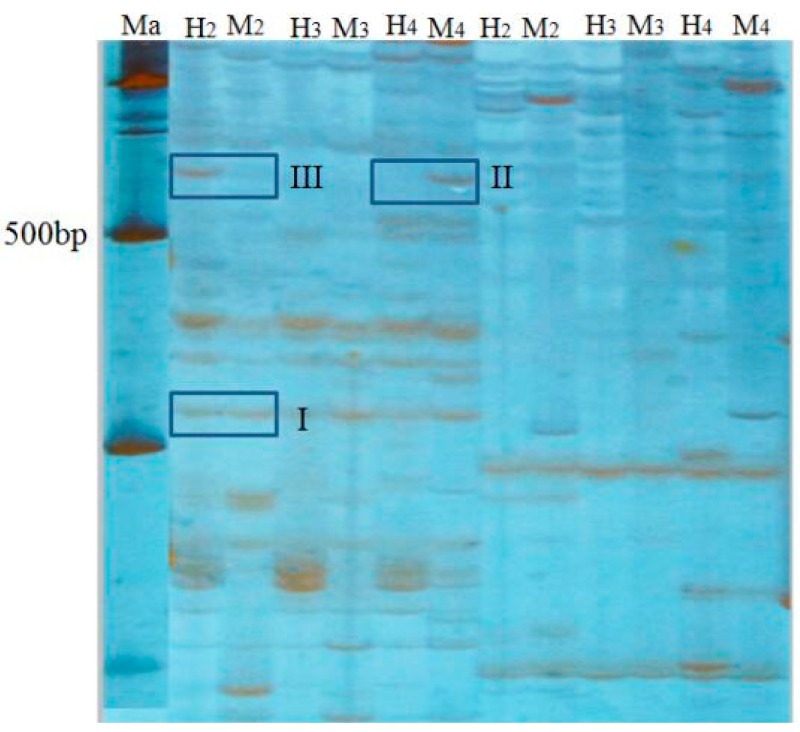
Genomic DNA MASP patterns of loach DNA of diploid (2*n*), triploid (3*n*) and tetraploid (4*n*) by primer combination E-CC and HM-TC. “H2”, “H3” and “H4”, respectively, indicate that total DNA of diploid (2*n*), triploid (3*n*) and tetraploid (4*n*) was digested by *Eco*R I/*Hpa* II; “M2”, “M3” and “M4”, respectively, indicate that total DNA of diploid (2*n*), triploid (3*n*) and tetraploid (4*n*) was digested by *Eco*R I/*Msp* I. “Ma” indicates the molecular weight marker (2000-bp ladder). I: Non-methylated sites (1.1); II: Full-methylated sites (0.1); III: Hemimethylated sites (1.0).

Genomic DNA of parents and offspring of different ploidy loach were cleaved by two groups of endonuclease, *EcoR* I/*Hpa* II denotes “H”, *EcoR* I/*Msp* I denotes “M”. This study chose eight pairs of selective amplification primers, and counted DNA bands on an electropherogram. At the same fragment size between different lanes of one polyacrylamide gel, a visible band was noted as “1”, no band was noted as “0”. Results counted the number of bands of different banding patterns between 100 to 700 bp of product that was amplified by eight pairs of selective primers. According to whether the amplification product bands appeared in the track, methylation band type was classified into four types (Table 7, Figure 2). Type I, both H and M tracks have bands, indicates that the site is non-methylation. Type II, M track has band and H track is without, indicates that the site is methylated inside the DNA double-strand, also known as full methylation. Type III, H track has band and M track is without, indicates that the site is methylated outside the DNA single strand, also known as hemimethylation. Type IV, neither H nor M track have bands, indicates three cases: that site methylation is outside the DNA double-strand, or is inside and outside the DNA double-strand, or the site has no CCGG sequence. The formulas of methylation ratios were as follows: full methylation ratios (%) = full methylation bands/total bands × 100%; hemimethylation ratios (%) = hemimethylation bands/total bands × 100%; total methylation ratios = full methylation ratios + hemimethylation ratios.

### 4.11. Test of Significance

The test used was the Duncan’s multiple range test by Statistical Product and Service Solutions (SPSS) 19.0 (IBM, Chicago, IL, USA). *p* < 0.05 means significant differences and *p* < 0.01 means extremely significant difference.

## Figures and Tables

**Table 1:** Analysis of genomic DNA methylation level and variance in different ploidy loach.

Ploidy	Total Sites	Non-Methylated Sites (%)	Methylated Sites (%; Mean ± SD)		
			Full Methylated Sites	Hemimethylated Sites	Total Methylated Sites
Diploid	2196	1291 (58.79 ± 6.68) ^a^	516 (23.50 ± 8.82) ^a^	389 (17.71 ± 6.62) ^a^	905 (41.21 ± 6.40) ^a^
Triploid	1684	1031 (61.22 ± 7.10) ^ab^	354 (21.02 ± 7.38) ^a^	299 (17.76 ± 5.66) ^a^	653 (38.78 ± 7.10) ^ab^
Tetraploid	2012	1267 (63.97 ± 6.23) ^b^	447 (22.22 ± 5.71) ^a^	298 (14.81 ± 4.48) ^a^	745 (37.03 ± 4.83) ^b^

Different small letters denote significant differences among different ploidy strains (*p* < 0.05).

**Table 2:** Comparison of DNA methylation patterns between triploid and tetraploid loach with diploid loach.

Patterns	3*n* or 4*n*		2*n*		3*n*-2*n*	4*n*-2*n*
	*Hpa II*	*Msp I*	*Hpa II*	*Msp I*	Number of Patterns and Frequency (%)	Number of Patterns and Frequency (%)
A					558 (26.94)	653 (31.82)
A1	1	1	1	1	359	390
A2	1	0	1	0	67	93
A3	0	1	0	1	132	170
B					493 (23.80)	550 (26.80)
B1	1	1	1	0	46	63
B2	1	1	0	1	67	95
B3	1	1	0	0	158	168
B4	1	0	0	0	104	104
B5	0	1	0	0	118	120
C					939 (45.34)	756 (36.84)
C1	1	0	1	1	77	65
C2	0	1	1	1	76	102
C3	0	0	1	1	285	220
C4	0	0	1	0	228	171
C5	0	0	0	1	273	198
D					81 (3.91)	93 (4.53)
D1	0	1	1	0	38	50
D2	1	0	0	1	43	43
B + C + D					1513 (73.05)	1399 (68.17)
Total					2071	2052

**Table 3:** Analysis of genomic DNA methylation level and variance in diploid and tetraploid loach and its hybrid F_1_ generation.

Hybrid Combinations		Total Sites	Non-Methylated Sites (%)	Methylated Sites (%; Mean ± SD)		
				Full Methylated Sites	Hemimethylated Sites	Total Methylated Sites
4 × 2 Obverse cross	Tetraploid (Female)	118	86 (72.88 ± 0.00) ^A^	19 (16.10 ± 0.00) ^A^	13 (11.02 ± 0.00) ^A^	32 (27.12 ± 0.00) ^A^
	Diploid (Male)	103	60 (58.25 ± 0.00) ^C^	23 (22.33 ± 0.00) ^B^	20 (19.42 ± 0.00) ^B^	43 (41.75 ± 0.00) ^C^
	Offspring Total	2101	1424 (67.78 ± 5.38) ^B^	445 (21.18 ± 5.400) ^B^	232 (11.04 ± 3.89) ^A^	677 (32.22 ± 5.38) ^B^
**Hybrid Combinations**		**Total Sites**	**Non-Methylated Sites (%)**	**Methylated Sites (%; Mean ± SD)**		
				**Full Methylated Sites**	**Hemi-Methylated Sites**	**Total Methylated Sites**
2 × 4 Inverse cross	Diploid (Female)	71	27 (38.03 ± 0.00) ^aA^	21 (29.58 ± 0.00) ^B^	23 (32.39 ± 0.00) ^B^	44 (61.97 ± 0.00) ^cB^
	Tetraploid (Male)	77	49 (61.25 ± 0.00) ^cB^	17 (21.25 ± 0.00) ^A^	14 (17.50 ± 0.00) ^A^	31 (38.75 ± 0.00) ^aA^
	Offspring Total	1476	809 (54.81 ± 8.93) ^bB^	355 (24.05 ± 6.04) ^A^	312 (21.14 ± 8.29) ^A^	667 (45.19 ± 9.06) ^bA^

Different small letters denote significant differences among different ploidy strains (*p* < 0.05). Different capital letters denote highly significant differences among different ploidy strains (*p* < 0.01).

**Table 4:** Analysis of genomic DNA methylation level and variance in obverse and inverse cross hybrid F_1_ generation of diploid and tetraploid loach.

Methylated Ratios	Obverse Cross (4 × 2) F_1_	Inverse Cross (2 × 4) F_1_
Full methylated ratios (%)	1.18 ± 5.40 ^a^	4.05 ± 6.04 ^A^
Hemimethylated ratios (%)	1.04 ± 3.89 ^a^	1.14 ± 8.29 ^B^
Total methylated ratios (%)	2.22 ± 5.38 ^a^	5.19 ± 9.06 ^B^

Different small letters denote significant differences among different ploidy strains (*p* < 0.05). Different capital letters denote highly significant differences among different ploidy strains (*p* < 0.01).

**Table 5:** Patterns of cytosine methylation in the female loach, male loach and their hybrid.

Patterns	Female		Male		Offspring		Difference Patterns Sites and Frequency (%)	
	*Hpa*II	*Msp*I	*Hpa*II	*Msp*I	*Hpa*II	*Msp*I	Obverse Cross (4 × 2)	Inverse Cross (2 × 4)
A							630 (30.41)	457 (27.17)
A1	+	+	+	+	+	+	405	353
A2	+	−	+	−	+	−	79	21
A3	−	+	−	+	−	+	51	6
A4	−	−	−	−	−	−	95	77
B							353 (17.04)	292 (17.36)
B1	+	+	−	+	+	+	91	65
B2	+	+	+	−	+	+	61	29
B3	−	+	+	+	+	+	62	37
B4	+	−	+	−	+	+	10	27
B5	−	+	−	−	+	+	3	3
B6	−	−	+	−	+	+	14	15
B7	−	−	−	+	+	+	4	14
B8	−	−	−	−	+	+	2	6
B9	+	+	−	−	+	+	90	70
B10	+	−	−	−	+	+	16	26
C							815 (39.33)	672 (39.95)
C1	−	−	−	+	−	−	75	73
C2	−	−	+	−	−	−	86	96
C3	−	+	−	−	−	−	61	54
C4	−	−	+	+	−	−	60	83
C5	+	+	−	−	−	−	88	79
C6	+	−	−	−	−	−	66	68
C7	+	+	+	−	−	−	26	13
C8	−	+	+	+	−	−	16	14
C9	−	+	−	+	−	−	43	21
C10	+	+	+	+	−	−	8	2
C11	+	+	−	+	−	−	31	16
C12	+	−	+	−	−	−	20	36
C13	−	+	+	+	−	+	51	32
C14	+	+	−	+	−	+	87	57
C15	+	+	+	−	+	−	40	14
C16	+	+	+	+	+	−	23	2
C17	+	+	−	−	+	−	2	2
C18	+	+	+	+	−	+	23	4
C19	+	+	−	+	+	−	7	3
C20	+	−	−	−	−	+	1	3
D							274 (13.22)	261 (15.52)
D1	−	−	+	+	+	+	12	10
D2	−	−	−	−	−	+	29	30
D3	−	−	−	−	+	−	13	15
D4	−	−	+	−	−	+	4	8
D5	−	−	+	+	−	+	7	13
D6	−	+	−	−	+	−	1	1
D7	−	+	+	+	+	−	5	2
D8	−	−	−	+	−	+	81	78
D9	−	−	+	−	+	−	28	23
D10	−	+	−	−	−	+	67	59
D11	+	−	−	−	+	−	27	22
B + C + D							1442 (69.59)	1225 (72.83)
Total							2072	1682

**Table 6:** Sequences of adaptors and primers used for methylation-sensitive amplified polymorphism (MSAP).

Primer/Adapter	Primer/Adapter	Sequences 5′–3′
*Eco*R I adapter	*Eco*R I adapter I	CTCGTAGACTGCGTACC
	*Eco*R I adapter II	AATTGGTACGCAGTC
*Hpa* II-*Msp* I adapter	*Hpa* II-*Msp* I adapter I	CGAGCAGGACTCATGA
	*Hpa* II-*Msp* I adapter II	GATCATGAGTCCTGCT
Preselective amplification primers	E-A	GACTGCGTACCAATTCA
	H-M	ATCCATGAGTCCTGCTCGGC
Selective amplification primers	E-CT	GACTGCGTACCAATTCACT
	E-CC	GACTGCGTACCAATTCACC
	HM-TC	ATCCATGAGTCCTGCTCGGCTC

**Table 7:** Methylation status of CCGG loci based differential sensitivity of isoschizomers.

Type	E + H Bands Type	E + M Bands Type	Status of CCGG Sites	Methylation Status of CCGG Sites
I	1	1	CCGG	Non-methylated
			GGCC	
II	0	1	C^5m^CGG	Methylation sites is inside the double-strand of DNA (Full methylated)
			C^5m^C^5m^GG	
III	1	0	^5m^CCGG	Methylation sites is outside the single-strand of DNA (Hemimethylated)
			C^5m^C^5m^GG	
IV	0	0	1. C^5m^C^5m^GG	1. Methylation site is inside and outside the double-strand of DNA
			GGC^5m^C^5m^	
			2. C^5m^CGG	2. Methylation site is outside the double-strand of DNA
			GGCC^5m^	
			3. Inexistence CCGG	3. Inexistence CCGG sequence
